# Complete Genome Characterization of Two Wild-Type Measles Viruses from Vietnamese Infants during the 2014 Outbreak

**DOI:** 10.1128/genomeA.00250-16

**Published:** 2016-04-14

**Authors:** Bas B. Oude Munnink, My V. T. Phan, Paul Kellam, Matthew Cotten, the VIZIONS Consortium

**Affiliations:** a Wellcome Trust Sanger Institute, Hinxton, United Kingdom; b Department of Infection, University College London, London, United Kingdom

## Abstract

A large measles virus outbreak occurred across Vietnam in 2014. We identified and obtained complete measles virus genomes in stool samples collected from two diarrheal pediatric patients in Dong Thap Province. These are the first complete genome sequences of circulating measles viruses in Vietnam during the 2014 measles outbreak.

## GENOME ANNOUNCEMENT

Measles virus is a member of the family *Paramyxoviridae* and causes measles, a highly contagious infectious disease. Measles mainly affects children age <5 years and was responsible for the deaths of 114,900 individuals in 2014 (1). Although measles vaccination is implemented in the Extended Programme for Immunization in Vietnam, measles virus infection is still prevalent in Vietnam, as demonstrated by a recent outbreak of measles virus in 2014 (2). During this outbreak, measles virus was estimated to have caused 30,000 infections, with 146 deaths (2). Until now, there has been only one report on the genome of a measles virus from Vietnam during this outbreak (3), with 2 short regions of the genome sequenced. No full measles virus genomes are available from Vietnam or Southeast Asia, leaving a large knowledge gap on the genetics of the circulating virus.

As part of a viral surveillance project in the Dong Thap Province (southern Vietnam) (4), a randomly primed deep-sequencing (agnostic sequencing) method (5) was used to document viral infections associated with diarrhea. Briefly, nucleic acid was purified from fecal samples, converted to double-stranded DNA (dsDNA) using random primers (6), and sequenced with Illumina HiSeq to generate 4 million 250-nucleotide (nt) paired-end reads per sample. Reads were trimmed from the 3′ end to a median Phred score of 35, with a minimum length of 175 nt, and *de novo* assembled using SPAdes version 3.5.0 (7), followed by improve_assembly (8). The work identified two complete wild-type measles virus genomes in stool samples from 2 diarrheal patients enrolled in 2014, during the period when measles was known to be circulating in Vietnam. The complete genomes of the virus isolates are named MVs/DongThap.VNM/06_14 (D8) (accession no. KU728742) and MVs/DongThap.VNM/08_14 (D8) (accession no. KU728743), in agreement with the WHO guidelines for naming measles virus strains (9). The average genome coverage of both viral genomes was 91- and 1,835-fold, respectively, demonstrating sufficient coverage to reliably assemble the complete genomes. Complete genome analysis revealed that the Vietnamese measles viruses share 99% nucleotide identity to the measles strain MVi/Muenchen.DEU/19.13[D8], the closest relative measles virus strain. The genomes have 9- and 19-nucleotide differences, respectively, compared to the measles strain MVi/Muenchen.DEU/19.13 (D8). No nucleotide changes were observed in the 450 nucleotides that encode the carboxy-terminal region of the nucleoprotein, the region used for genotyping.

Both patients were admitted to the Provincial Hospital of Dong Thap due to acute diarrhea, and the generation of whole measles virus genomes from these patients suggests that the episodes of diarrhea reported in these patients may be a consequence of measles virus infection, consistent with previous observations (10). In addition, these patients were 7 and 8 months of age, demonstrating that children can be infected with measles virus before reaching the age of vaccination (9 months). In conclusion, we report the first 2 whole genomes of wild-type measles virus genotype D8 during the 2014 outbreak in Vietnam. The viral genomes belonged to measles virus genogroup D8 and will provide a useful reference for measles surveillance in the region.

### Nucleotide sequence accession numbers.

The complete genome sequences of the MVs/DongThap.VNM/06_14 (D8) and the MVs/DongThap.VNM/08_14 (D8) isolates are available at GenBank under the accession numbers KU728742 and KU728743.
